# Neuregulin 4 as a novel adipokine in energy metabolism

**DOI:** 10.3389/fphys.2022.1106380

**Published:** 2023-01-10

**Authors:** Yuanbin Liu, Mingkai Chen

**Affiliations:** Department of Gastroenterology, Renmin Hospital of Wuhan University, Wuhan, Hubei, China

**Keywords:** neuregulin, metabolism, adipokine, thermogenesis, energy

## Abstract

Adipose tissue has been shown to play a key role in energy metabolism and it has been shown to regulate metabolic homeostasis through the secretion of adipokines. Neuregulin 4 (Nrg4), a novel adipokine secreted mainly by brown adipose tissue (BAT), has recently been characterized as having an important effect on the regulation of energy homeostasis and glucolipid metabolism. Nrg4 can modulate BAT-related thermogenesis by increasing sympathetic innervation of adipose tissue and therefore has potential metabolic benefits. Nrg4 improves metabolic dysregulation in various metabolic diseases such as insulin resistance, obesity, non-alcoholic fatty liver disease, and diabetes through several mechanisms such as anti-inflammation, autophagy regulation, pro-angiogenesis, and lipid metabolism normalization. However, inconsistent findings are found regarding the effects of Nrg4 on metabolic diseases in clinical settings, and this heterogeneity needs to be further clarified by future studies. The potential metabolic protective effect of Nrg4 suggests that it may be a promising endocrine therapeutic target.

## Introduction

The prevalence and incidence of metabolic disorders such as insulin resistance (IR) (Saklayen, 2018), diabetes (Saeedi et al., 2019), obesity (Chooi et al., 2019), and non-alcoholic fatty liver disease (NAFLD) (Riazi et al., 2022) are increasing globally, posing substantial public health concerns and burdens. However, there is still a lack of effective treatment approaches for these metabolic disorders, especially for NAFLD. Considerable potential remains for the development of therapeutics to target these metabolic diseases. Accordingly, it is of great importance to identify novel promising targets and related pathways against the global epidemic.

Neuregulins (Nrgs) are multipotent polypeptide growth factors and members of the epidermal growth factor family, which can activate members of the ErbB family of receptor tyrosine kinases as ligands (Geissler et al., 2020; Tutunchi et al., 2020). Currently, four structurally related Nrgs (Nrg1-4) have been identified, which are implicated in various biological processes (Falls, 2003). Nrg1 can bind to both ErbB3 and ErbB4 and its roles in cardiovascular disease (Odiete et al., 2012), neurodevelopmental disorders (Shi and Bergson, 2020), and cancers (Zhang et al., 2022a) are well recognized. Nrg2 (Carraway et al., 1997) and Nrg3 (Zhang et al., 1997) was first identified as a ligand for ErbB3/ErbB4 and ErbB4 respectively in 1997, which is mainly enriched in neural tissue. Nrg4 was identified as the fourth Nrg in 1999 (Harari et al., 1999) and functions favorably *via* ErbB4. Nrg4 exerts a major role in glycolipid metabolism and energy homeostasis as an adipokine enriched in brown adipose tissue (BAT). (Gavaldà-Navarro et al., 2022).

Modulating adipokines, which act as crosstalk messengers between adipose tissue and other organs, has recently provided new insights into the pathogenesis of metabolic diseases and may have treatment prospects (Cheng and Yu, 2022; Francisco et al., 2022; Kim et al., 2022). In recent years, the role of Nrg4 as a novel signaling protein in metabolic derangements have been revealed and may serve as a novel therapeutic candidate. The NRG/ErbB signaling pathway is currently a promising target for areas related to metabolism. Therefore, we summarize here the mechanisms underlying the actions of Nrg4 in metabolism and their effects on diverse metabolic diseases in preclinical and clinical settings and suggest possible directions for future investigations.

### The role of BAT in metabolism

Adipose tissue has a vital role in energy metabolism and homeostasis and adipose tissue dysfunction have been proven to lead to metabolic diseases such as obesity and diabetes (Torres Irizarry et al., 2022). Adipose tissue is traditionally divided into white adipose tissue (WAT) and BAT, and recently a new type of thermogenic adipocyte, the beige adipocyte, has been proposed (Wu et al., 2012). WAT contains a single large lipid droplet and fewer blood vessels than BAT, whereas BAT has numerous small lipid droplets and much higher mitochondrial content (Saely et al., 2012). The BAT is distributed throughout the body and the content of brown adipocytes gradually decreases with age. Functionally, the WAT is primarily responsible for storing excess energy in the form of triglycerides; while by increasing energy metabolism and thermogenesis, beige adipose tissue/BAT plays a crucial role in regulating glycolipid metabolism (Wang et al., 2022a). However, the beige adipose tissue rarely expressed thermogenic genes in the absence of stimulation and developed similar levels of thermogenic gene expression to brown adipocytes under stimulation (Wu et al., 2012).

BAT has historically been recognized as a major site of non-shivering thermogenesis. Recently, the BAT has gradually been understood as a secretory organ that can secrete a series of cytokines, which are called ‘batokines’ and thus mediate crosstalk with other organs (Villarroya et al., 2017; Villarroya et al., 2019). These brown adipokines can exert local autocrine and paracrine effects thereby mediating thermogenesis in brown adipocytes, as well as directed endocrine effects on distant tissues and organs (Gavaldà-Navarro et al., 2022). Multiple agents have been identified as endocrine factors, including but not limited to fibroblast growth factor-21 (FGF21), interleukin-6 (IL-6), some microRNAs, and the presently referenced Nrg4 (Gavaldà-Navarro et al., 2022).

Among the organs that commonly interact with the BAT in an endocrine manner are the liver, heart, and skeletal muscle. These batokines including Nrg4 have been shown to improve metabolic diseases such as NAFLD, atherosclerosis, and diabetes (Cereijo et al., 2020; Spann et al., 2021; Liu et al., 2022). In addition to organs, several tissues are targeted by BAT following thermogenic stress such as WAT, sympathetic neurons, and immune cells, contributing to tissue remodeling (Villarroya et al., 2019). In response to stimuli such as cold, some WATs may undergo ‘browning’, leading to the conversion to beige adipose tissues (Wang and Seale, 2016). The batokines have been revealed to facilitate the adaptation of BAT to thermogenic stimuli such as brown adipocyte hypertrophy, hyperplasia, angiogenesis, and innervation in an autocrine and paracrine manner during this process (Villarroya et al., 2017). These beige adipose tissues may improve metabolic disorders and insulin sensitivity by consuming energy through thermogenesis, thus modulating the browning process may be a promising option for the treatment of metabolic diseases (Betz and Enerbäck, 2018). A number of batokines including Nrg4 can deliver paracrine effects by targeting sympathetic neurons and forming brown adipocyte-nerve cell signaling pathways (Gavaldà-Navarro et al., 2022). This signaling potentially acts in mediating BAT thermogenesis (Christian, 2015; Zeng et al., 2019; Hu et al., 2020). Furthermore, some batokines can target immune cells and thus exert anti-inflammatory effects (Cereijo et al., 2018), and activation of BAT has also been linked to the suppression of the local pro-inflammatory environment (Villarroya et al., 2018). Therefore, BAT-secreted batokines may be potential targets for the treatment of some metabolic diseases by mediating crosstalk between BAT and other organs and tissues.

### Nrg4 potentially regulates thermogenesis

Wang et al. (Wang et al., 2014) explored the significance of Nrg4 in defense against excessive cold stimulation. By secretome analysis of 12 mouse tissues, the authors identified 26 gene clusters including Nrg4 that were enriched in BAT and induced during brown adipocyte differentiation (Wang et al., 2014). Nrg4 was abundantly expressed in BAT and at lower levels in WAT (Wang et al., 2014). In addition, its expression was relatively low in other tissues, including skeletal muscle, liver, brain, and heart (Wang et al., 2014). Not surprisingly, Nrg4 expression was elevated in BAT due to acute cold exposure (Wang et al., 2014). However, the role of Nrg4 in cold-stimulated BAT thermogenesis appeared not to be indispensable as demonstrated by the construction of Nrg4-deficient mouse models. Wild-type and Nrg4-null mice had similar rectal body temperatures, cold exposure-induced uncoupling protein-1 (UCP1) and deiodinase two expressions, and plasma insulin, lipocalin, and leptin levels (Wang et al., 2014). The results seemed to suggest that Nrg4 may not be directly involved in BAT thermogenesis but may act indirectly by influencing other tissues.

BAT thermogenesis has been demonstrated to be highly dependent on the sympathetic nervous system (SNS) and the control of BAT thermogenesis is ensured by the central nervous system. The brain regions involved in the regulation of body temperature and the regulation of energy balance are implicated in the central regulation of BAT thermogenesis. Many hypothalamic nuclei including the arcuate nucleus, preoptic area, dorsomedial hypothalamus, paraventricular hypothalamus, lateral hypothalamus, and ventral medial hypothalamus are responsible for the control of thermogenesis (Labbé et al., 2015). SNS-mediated BAT activity is controlled by various neuronal circuits in brain regions involved in homeostatic regulation. Activation of BAT thermogenesis traditionally involves the release of norepinephrine from the sympathetic nervous system following cold exposure, which acts on β3-adrenergic receptors and thus induces adrenergic signaling, leading to activation of thermogenic programs and browning (Tabuchi and Sul, 2021). Nrg4 has been characterized to play an essential role in regulating the growth and elaboration of neural dendrites within the central nervous system through an autocrine/paracrine approach since it was first identified as a regulator of neuronal development (Paramo et al., 2018; Paramo et al., 2019; Paramo et al., 2021). Rosell et al. (Rosell et al., 2014) analyzed the gene expression of interscapular BAT, inguinal subcutaneous WAT, and visceral mesenteric WAT under warm (28 °C) or cold (6 °C) conditions in mice by transcriptomic studies. The authors aimed to elicit the characteristics of a ‘brown in white’ transcription signature in WAT by microarray analysis, i.e., differentially expressed genes and related pathways that may be associated with adipocyte browning. Nrg4 was one of the five genes identified by the microarray and was secreted mainly by mature adipocytes that promote neurite growth. By Nrg4 knockdown (Nrg4KD), neurite growth in the brown adipocyte-specific medium was observed to be restricted (Rosell et al., 2014). Thus, Nrg4 was proposed to possibly function through adipocyte-nerve cell signaling, thereby promoting innervation of BAT (Christian, 2015). However, the effects of Nrg4 on thermogenesis may not be limited to involvement in adipocyte-neuron interaction, but may also affect WAT browning employing autocrine/paracrine or influence adipocyte numbers and preadipocyte differentiation (Christian, 2015). Future studies to fully clarify more mechanisms of Nrg4 in BAT thermogenesis are needed.

**FIGURE 1:**
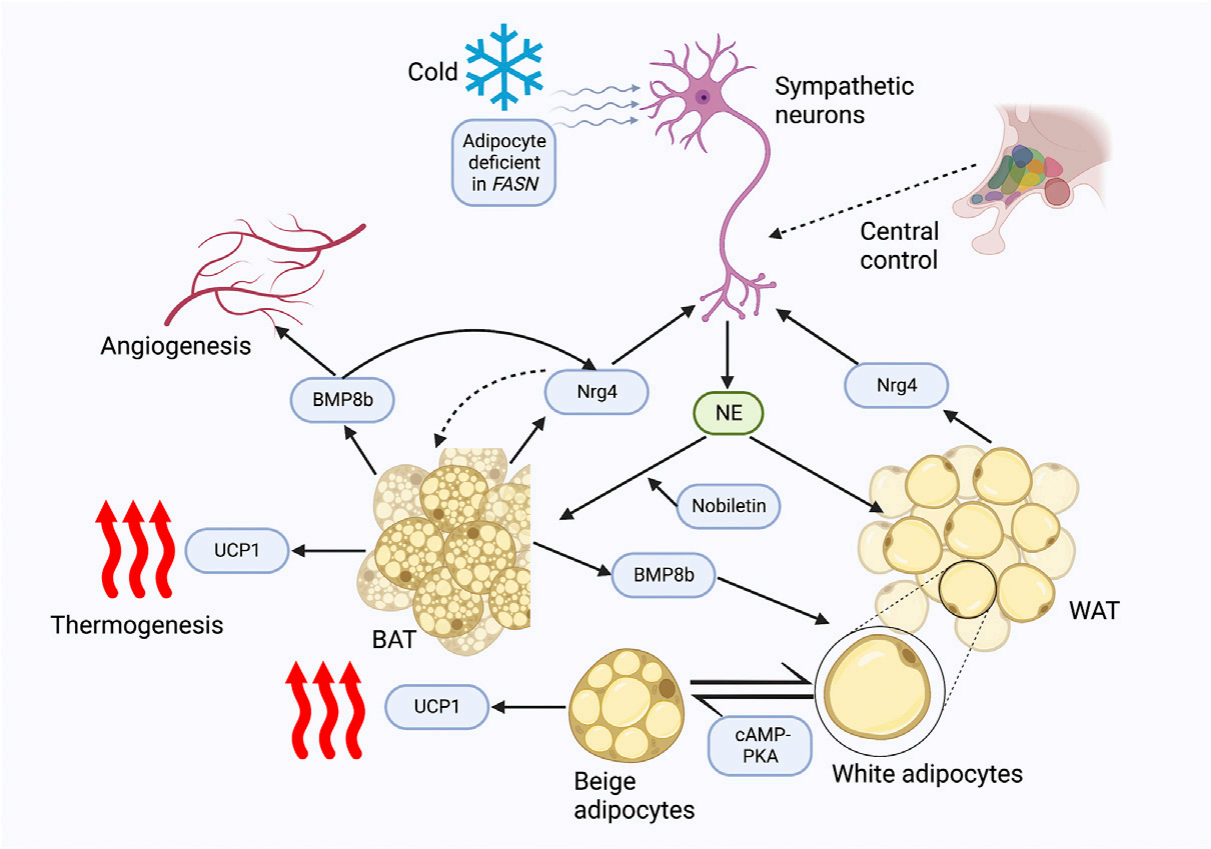
Nrg4 potentially regulates thermogenesis. Both cold and FASN deficiency promote sympathetic activation of BAT thermogenesis and Nrg4 production; FASN deficiency is independent of sympathetic neuron and Nrg4, whereas cold is dependent on sympathetic activation and Nrg4 for optimal thermogenesis. Sympathetic neurons release NE to activate thermogenesis and WAT browning, while Nrg4, mainly secreted by BAT, promotes neurite growth, and thus increases adipose tissue innervation. BMP8b secreted by BAT can increase innervation of adipose tissue by promoting Nrg4 expression and promote angiogenesis, which can lead to neurovascular remodeling. The cAMP-PKA pathway is involved in the browning of white adipocytes. A natural compound, nobiletin, promotes β-adrenergic stimulation to increase Nrg4 expression in *in vitro* experiments. Abbreviations: FASN, fatty acid synthase; BAT, brown adipose tissue; NE, norepinephrine; Nrg4, neuregulin four; BMP8b, bone morphogenetic protein 8b; UCP1, uncoupling protein-1; WAT, white adipose tissue; cAMP-PKA, cyclic AMP/protein kinase A.

Additional insights into the role of Nrg4 in BAT thermogenesis have been recently presented. Bone morphogenetic protein 8b (BMP8b) is primarily expressed in mature brown adipocytes and has been shown to potentiate BAT thermogenesis by sensitizing brown adipocytes to adrenergic inputs (Whittle et al., 2012). Pellegrinelli et al. (Pellegrinelli et al., 2018) revealed the peripheral effects of BMP8b in thermogenesis by constructing adipose tissue BMP8b overexpressing transgenic mice. BMP8b overexpressing mice demonstrated enhanced WAT browning and more robust beige lipid thermogenesis (Pellegrinelli et al., 2018). BMP8b overexpressing mice exhibited elevated innervation density of BAT and subcutaneous white fat, although in *in vitro* assays it did not promote sympathetic axon branch growth (Pellegrinelli et al., 2018). Alternatively, BMP8b can modulate Nrg4 function at both the transcriptional and post-transcriptional levels, and increased Nrg4 expression by BMP8b has also been shown in this study to increase sympathetic innervation of adipose tissue by promoting the branching and growth of sympathetic axons (Pellegrinelli et al., 2018). In addition to the regulation of adipose tissue innervation, BMP8b also induced vascular remodeling by increasing angiogenic factors in activated adipocytes (Pellegrinelli et al., 2018). These interesting findings demonstrated that BMP8b functioned to promote adipose tissue thermogenesis by inducing neurovascular remodeling, in which the interconnection between Nrg4 and BMP8b played an essential role. Another study found that a deficiency of fatty acid synthase (FASN) can mimic cold-induced thermogenic signals (Henriques et al., 2020). Interestingly, both cold and adipocyte deficient in FASN (iAdFASNKO)-induced signaling increased sympathetic activity as well as Nrg4 expression, but thermogenesis induced by iAdFASNKO was shown to be independent of sympathetic innervation and Nrg4, as neither inguinal WAT denervation nor Nrg4 loss in iAdFASNKO mice attenuated adipocyte browning (Henriques et al., 2020). In contrast, cold-induced signaling was revealed to require sympathetic signaling and the involvement of Nrg4, as UCP1 expression levels were reduced by approximately 50% in Nrg4 whole-body knockout mice and WAT browning was attenuated (Henriques et al., 2020). Furthermore, it was noted that both thermogenic signals require cyclic AMP/protein kinase A (cAMP-PKA) signaling in adipocytes to induce browning (Henriques et al., 2020). A further study used a constructed plasmid to enhance the expression of the *NRG4* gene and found that mice injected with the plasmid had a 1.0 °C increase in body temperature after 1 day, which was maintained for 2 weeks. In addition, Nrg4 overexpression enhanced the expression of BAT thermogenic genes including UCP1 (Ma et al., 2016). These studies illustrated the relevance of Nrg4 in cold-induced optimal thermogenesis, which conflicted with the results obtained by Wang et al. (Wang et al., 2014). This may be explained by the different duration and temperature of cold exposure (6 days of exposure at 6 °C (Henriques et al., 2020) vs acute cold exposure at 4 °C (Wang et al., 2014)), however, the plausibility of such factors needs to be demonstrated by future *in vivo* studies. Nobiletin, a natural compound found in citrus fruits, was shown to induce Nrg4 and FGF21 expressions by enhancing β-adrenergic stimulation in brown adipocytes *in vitro* (Kihara-Negishi et al., 2022). However, future *in vivo* experiments are needed to determine its potential effect in regulating Nrg4-mediated thermogenesis (Figure 1).

The WAT browning effect of Nrg4 was also confirmed in clinical cohorts. A study included two independent patient cohorts to explore the relationship between Nrg4 and thermogenic markers in subcutaneous adipose tissue (SAT) and visceral adipose tissue (VAT) (Comas et al., 2019). Nrg4 expression was shown to be positively correlated with the expression of thermogenic genes including UCP1 (Comas et al., 2019). Interestingly, transmembrane protein 26, a selective surface protein marker of beige adipocytes, was identified as the optimal predictor of Nrg4 expression in adipose tissue in a multiple linear regression analysis (Comas et al., 2019). Accordingly, this study revealed that Nrg4 is a novel marker of beige adipocytes.

### Nrg4 and IR

Nrg4-ErbB4 signaling can regulate islet endocrine cell differentiation and development in *ex vivo* experiments (Huotari et al., 2002). Nrg4 was similarly shown to be the most insulin-stimulating of the Nrg family in a rat insulinoma cell line in a subsequent study, suggesting that Nrg4 may have a potent role in regulating islet cell growth and secretion (South et al., 2013).

The inhibitory effect of Nrg4 on inflammation has been previously reported. Reduced Nrg4 levels were observed in mice and human samples with inflammatory bowel disease, and Nrg4 treatment blocked inflammatory cytokine-induced apoptosis of colonic epithelial cells both *in vivo* and *in vitro*, which demonstrated the potential anti-inflammatory effect of Nrg4-ErbB4 signaling (Bernard et al., 2012; McElroy et al., 2014). Chronic low-grade tissue inflammation in obese subjects is well-established to be strongly associated with IR (Gasmi et al., 2021). *NRG4* gene transfer *via* recombinant plasmids was revealed to inhibit chronic inflammation of WAT in high-fat diet (HFD)-induced obese mice by reducing macrophage infiltration in adipose tissues, thereby improving insulin sensitivity (Ma et al., 2016). This is consistent with a subsequent study, which demonstrated that Nrg4 mRNA expression levels were negatively correlated with blood glucose and plasma insulin concentrations, and Nrg4 expression *in vitro* was regulated by inflammatory signaling stimuli, as evidenced by pro-inflammatory and anti-inflammatory actions exhibiting opposite changes (Chen et al., 2017a). Following a hyperinsulinemic-euglycemic clamp study, the authors found that Nrg4 transgenic mice increased insulin sensitivity by enhancing peripheral glucose metabolism, thereby regulating systemic glucose metabolism (Chen et al., 2017a). Notably, this augmented effect was attributed to increased glucose metabolism in peripheral organs, not in the liver. Achieving this effect requires intact Nrg4-ErbB4 signaling, as Nrg4 did not increase glucose uptake in the adipocytes of ErbB4-deficient mice in one study (Zeng et al., 2018). Consistently, Nrg4 expression was also shown to be negatively correlated with the expression levels of inflammatory cytokines in adipose tissue from clinical patients (Comas et al., 2019).

Hepatic steatosis is known to be closely linked to IR (Lee et al., 2022). A study analyzed the potential of adipose tissue-derived mesenchymal stem cell (ADSC) and ADSC overexpressing Nrg4 (Nrg4-ADSC) to improve IR in HFD-induced obese mice. Both ADSC and Nrg4-ADSC were shown to improve IR by reducing pro-inflammatory cytokine levels and upregulating the expression of glucose transporter 4 (GLUT4), whereas Nrg4-ADSC was more effective in improving disturbed glucose metabolism in obese mice (Wang et al., 2019a). Further work revealed that Nrg4-ADSC could enhance this beneficial effect by reducing liver lipogenesis, whereas ADSC did not demonstrate this effect, suggesting that the mechanism by which Nrg4 improves IR can be achieved in part by limiting liver steatosis (Wang et al., 2019a).

**FIGURE 2:**
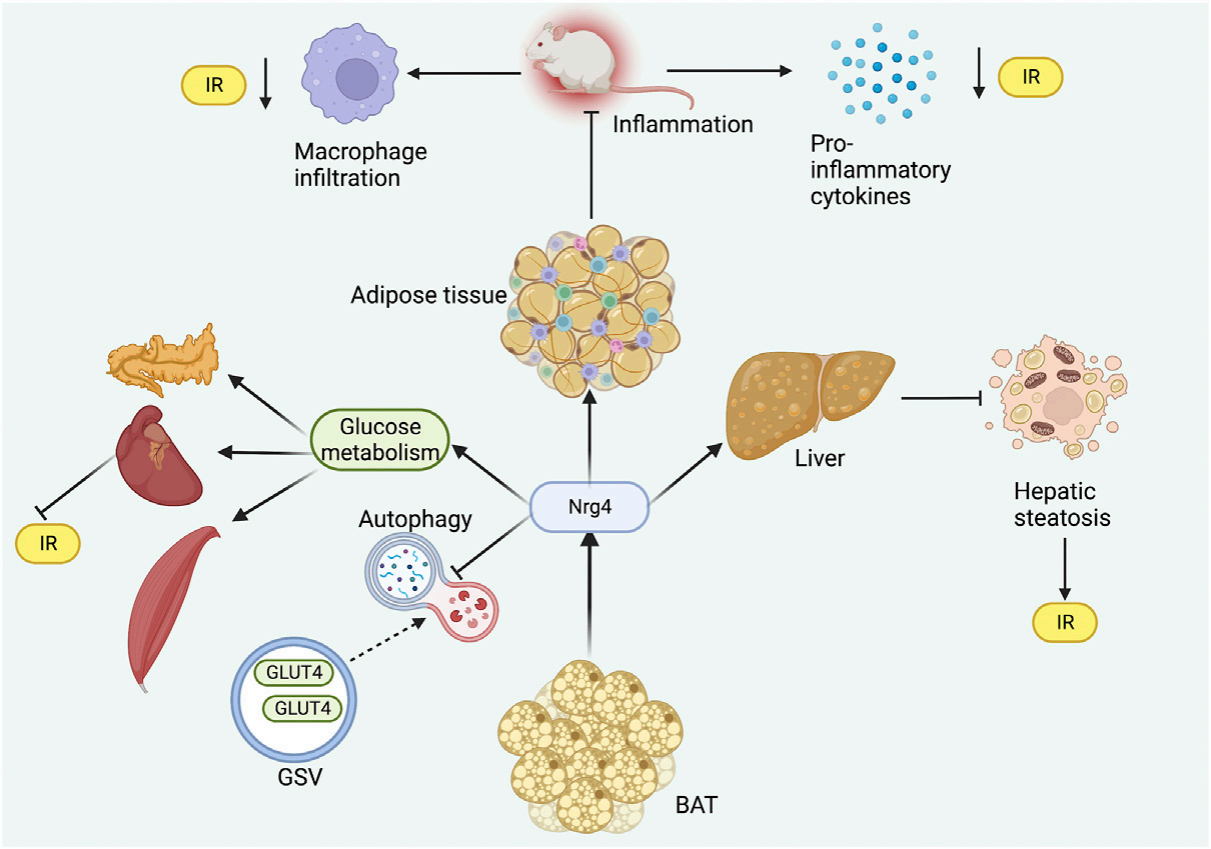
Role of Nrg4 in IR. Nrg4 ameliorates inflammation in adipose tissue by reducing macrophage infiltration and pro-inflammatory cytokine levels. Nrg4 improves systemic energy metabolism by increasing glucose metabolism in peripheral organs, while reducing autophagy in GSV. Nrg4 also improves IR by improving hepatic steatosis. Abbreviations: Nrg4, neuregulin four; BAT, brown adipose tissue; IR, insulin resistance; GLUT4, glucose transporter four; GSV, GLUT4 storage vesicle.

Autophagy dysregulation is a major contributing pathogenic mechanism in metabolic diseases (Kitada and Koya, 2021). Another study demonstrated by *in vitro* experiments that Nrg4KD adipocytes exhibited IR and demonstrated reduced insulin receptor (InsR) expressions and GLUT4 protein levels (Díaz-Sáez et al., 2021). Similarly, inflammatory signaling was enhanced in Nrg4KD adipocytes, and Nrg4 treatment could reduce inflammation and restore InsR and GLUT4 protein expression (Díaz-Sáez et al., 2021). Further anti-inflammatory treatment with sodium salicylate and dexamethasone applied to Nrg4KD adipocytes was revealed to reverse the expression of InsR but had no restorative effect on GLUT4 protein (Díaz-Sáez et al., 2021). Therefore, there may be additional mechanisms mediating the changes in GLUT4 protein content. The authors further found decreased levels of GLUT4 storage vesicle (GSV) protein in Nrg4KD adipocytes, thus speculating that this may be due to autophagic degradation. As expected, the mechanistic target of rapamycin complex 1 (mTORc1), a marker of autophagy inhibition, was found to be reduced in Nrg4KD adipocytes (Díaz-Sáez et al., 2021). In addition, several autophagy-associated markers were also noted to increase (Díaz-Sáez et al., 2021). Blocking autophagy recovered Glut4 and GSV protein profiles, demonstrating that Nrg4 can improve glucose metabolism and IR at least *in vitro* by reducing autophagy (Figure 2).

Despite the beneficial effects of Nrg4 on IR under experimental conditions, it seemed to exhibit the opposite detrimental action in a human study. Nrg4 was negatively correlated with insulin sensitivity in humans confirmed by the hyperinsulinemic-euglycemic clamp study (Martínez et al., 2022), contrary to the findings of another human study (Comas et al., 2019). Moreover, they found that human recombinant Nrg4 in the human HepG2 cell line could impair mitochondrial respiration but showed no effect on the gene expression involved in lipid metabolism (Martínez et al., 2022). These findings were inconsistent with the results derived from animal experiments, suggesting that there were other factors influencing the human study of Nrg4. It is noted by the authors that the different stages of diabetes and the lack of validation at the protein level may have led to conflicting conclusions (Martínez et al., 2022). Furthermore, *in vitro* experiments for this study should be performed in human primary hepatocytes due to the enhanced glycolytic pathway shown in the HepG2 cell line (Martínez et al., 2022). However, plausible explanations for these contradictory results are lacking, and more well-designed studies exploring the underlying reasons are needed in the future.

### Nrg4 and obesity

Adipose tissue Nrg4 mRNA expression level has been demonstrated to be decreased in both mice and humans with obesity, suggesting a possible obesity-protective role (Wang et al., 2014). Since obesity is characterized by chronic inflammatory infiltration of adipose tissue, pro-inflammatory signaling may similarly contribute to the reduced Nrg4 expression of adipose tissue in obesity. *In vitro* treatment of adipocytes with tumor necrosis factor-alpha (TNFα) and interleukin 1β (IL-1β) revealed a decrease in Nrg4 expression, which supported this possibility (Wang et al., 2014). Nrg4 was regulated by upstream inflammatory signals and drove altered expression, in parallel Nrg4 was noted to downregulate inflammatory gene mRNA levels in epididymal WAT (Chen et al., 2017a). Interestingly, Nrg4 overexpression prevented obesity by reducing adipose tissue inflammation but did not affect pre-existing obesity and body weight (Ma et al., 2016).

Another study further revealed the regulatory mechanism of Nrg4 in obese mice. Increased energy expenditure and fuel oxidation and lower leptin levels were demonstrated in Nrg4 transgenic mice, suggesting that Nrg4 could promote energy metabolism in a catabolic direction (Chen et al., 2017a). In addition, Nrg4 transgenic mice were shown to increase mRNA expression of several adipokines, including adipsin, adiponectin, and vascular endothelial growth factor α (VEGFα) (Chen et al., 2017a). These adipokines have been characterized as exhibiting effects that can ameliorate metabolic dysregulation in obesity (Achari and Jain, 2017; Milek et al., 2022; Zhou et al., 2022), suggesting that Nrg4 overexpression can further contribute to the normalization of the adipokine profile. These anti-obesity effects also necessitated the presence of ErbB4, as ErbB4 deficiency led to severe inflammation, increased lipogenesis, and increased serum leptin levels (Zeng et al., 2018).

**FIGURE 3:**
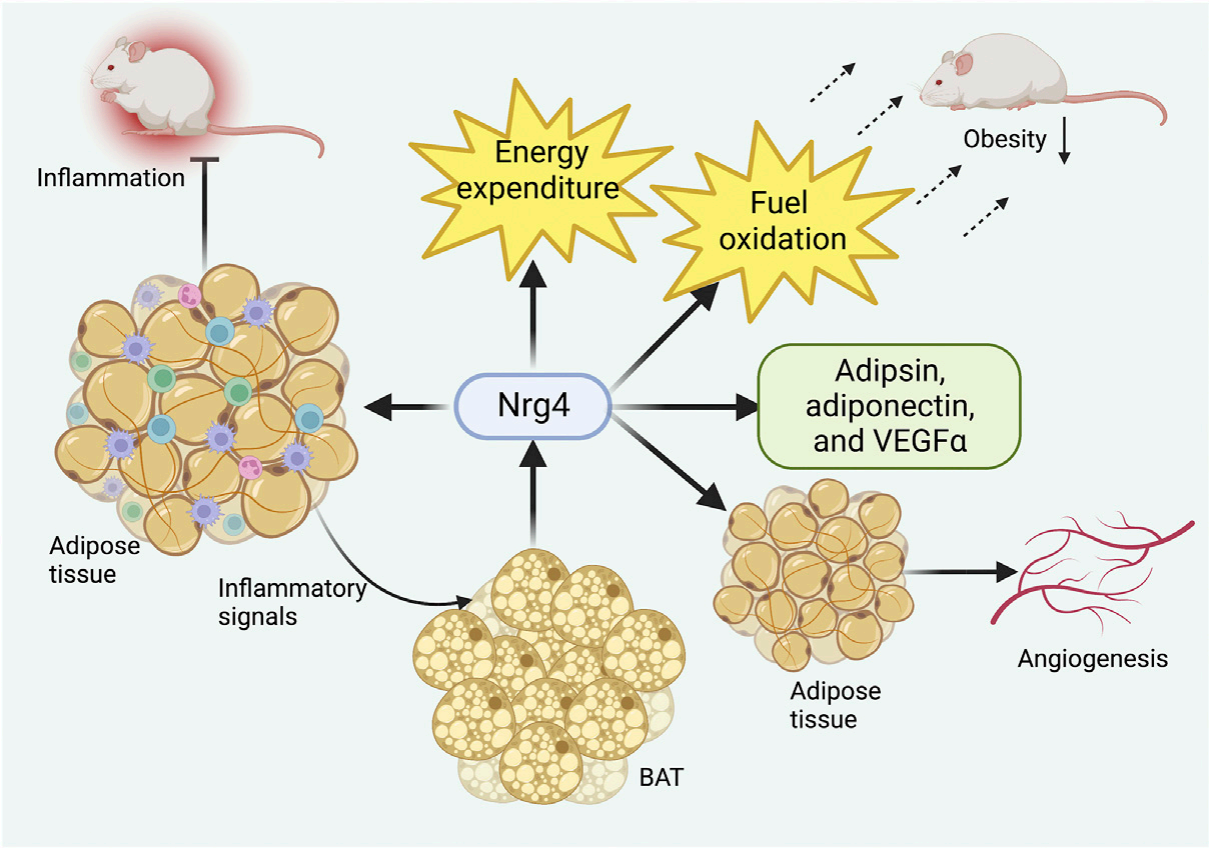
Role of Nrg4 in obesity. Chronic inflammation of adipose tissue can activate BAT to produce Nrg4, which in turn ameliorates adipose tissue inflammatory infiltration. Nrg4 can promote energy metabolism by increasing energy expenditure and fuel oxidation, together with facilitating the production of metabolically beneficial adipokines. Finally, Nrg4 can improve obesity by promoting adipose tissue angiogenesis. Abbreviations: Nrg4, neuregulin four; BAT, brown adipose tissue; VEGFα, vascular endothelial growth factor α.

Nrg4 has been demonstrated to improve obesity and related metabolic disorders by increasing adipose tissue angiogenesis as a pro-angiogenic factor. Nrg4 was revealed to activate adipose tissue angiogenesis *in vivo* and *in vitro*, and genetic deletion of Nrg4 can lead to reduced vascularity in BAT and WAT resulting in obesity even under a normal diet (Nugroho et al., 2018a). The beneficial metabolic effects of Nrg4 were observed to be eliminated by anti-angiogenic drug treatment in another study (Nugroho et al., 2018b), suggesting that the angiogenic effect of Nrg4 was an essential strategy in preventing obesity (Figure 3).

A cross-sectional study that included 1,212 patients with obesity found significantly lower serum Nrg4 levels in patients with metabolic syndrome (MetS) compared to normal controls, and circulating Nrg4 levels were negatively correlated with waist circumference and body mass index (BMI), although no significant differences in Nrg4 expression were found in overweight/obese patients (Cai et al., 2016). These results demonstrated that circulating Nrg4 levels were similarly reduced in obesity, consistent with findings in adipose tissue (Comas et al., 2019; Wang et al., 2020), suggesting that Nrg4 deficiency may be an important feature of obesity. Another recent study including adults with obesity of the same community origin further identified that both circulating Nrg4 and adipsin levels were significantly associated with waist circumference, visceral fat, and MetS, providing additional clinical evidence for the association of Nrg4 with obesity (Guo et al., 2021).

Physical activity is a well-recognized lifestyle intervention to combat obesity (Oppert et al., 2021). Nrg4 levels in men with obesity were significantly increased by 12 weeks of resistance training (conventional, circular, and interval) (Alizadeh et al., 2021), suggesting that Nrg4 may be involved in exercise-induced weight loss. Another study examined the effects of three different training modalities, high-intensity interval training (HIIT), circuit resistance training (CRT), and moderate-intensity continuous training (MICT), on serum Nrg4 levels and various metabolic-related parameters in men with obesity. Increased Nrg4 levels were significantly associated with improved serum glycolipid metabolism markers (Saeidi et al., 2021). The results showed that all three modalities increased serum Nrg4 levels and showed favorable metabolic regulatory effects, while the HIIT and CRT programs exerted greater effects on Nrg4 and metabolic risk factors than the MICT program (Saeidi et al., 2021). These findings suggest that physical activity may improve dysregulated metabolic status, in part by increasing Nrg4 levels.

### Nrg4 to liver signaling

NAFLD is the most common chronic liver disease worldwide and can range from simple steatosis to more severe non-alcoholic steatohepatitis (NASH), which can eventually lead to cirrhosis and hepatocellular carcinoma (HCC) (Pouwels et al., 2022). The occurrence and development of NAFLD are currently embraced by the ‘multiple hit’ theory, which refers to a combination of contributing factors that lead to the pathogenesis of NAFLD (Buzzetti et al., 2016). Dietary preferences, environmental factors, and genetic predisposition contribute to the development of IR, obesity, and intestinal dysbiosis, which consequently leads to impaired lipid metabolism and lipotoxicity, inducing pathophysiological changes such as oxidative stress, mitochondrial dysfunction, endoplasmic reticulum stress, and cell death (Buzzetti et al., 2016; Friedman et al., 2018; Manne et al., 2018). Adipose tissue dysfunction and liver lipid metabolism disorders have been revealed as major hallmarks of the pathogenesis of NAFLD, such as adipokine dysregulation, excessive fatty acid uptake, and abnormal lipogenesis (Manne et al., 2018; Azzu et al., 2020).

Wang et al. (Wang et al., 2014) identified the tissue binding site of Nrg4 by employing secreted alkaline phosphatase to form a fusion protein with the extracellular fragment of the Nrg4 protein. In contrast to sites such as the heart, skeletal muscle, and BAT, Nrg4 exhibited strong binding activity in the liver and was mediated through ErbB3 and ErbB4 receptors (Wang et al., 2014). These important findings suggested that Nrg4 could act by directly and specifically binding to hepatic ErbB receptors.

It is widely recognized that IR is closely correlated with liver metabolic disturbances such as NAFLD (Watt et al., 2019; Marušić et al., 2021). Nrg4 expression in BAT was identified negatively correlated with hepatic triglyceride levels (Weitkunat et al., 2017). Nrg4 deficiency exacerbated IR and diet-induced obesity, but by liver-specific knockout of Nrg4, serum and liver triglyceride levels and hepatic gene expression did not differ from WT mice (Wang et al., 2014). However, the researchers observed significantly increased expression of several genes associated with *de novo* lipogenesis including glucokinase, malic enzyme, fatty acid synthase, stearoyl coenzyme A desaturase 1, and ELOVL fatty acid elongase 5 (Wang et al., 2014). The mRNA level of a key transcription factor sterol regulatory element binding protein-1c (SREBP-1c) and protein levels of the precursor SREBP-1 were also notably increased in the liver (Wang et al., 2014). Notably, Nrg4/ErbB4 activation was further characterized as impacting *de novo* lipogenesis in a cell-autonomous manner and directly regulating SREBP-1c in response to activation of the liver-X receptor (Wang et al., 2014). Finally, Nrg4 transgenic mice significantly improved lipid metabolism disorders and hepatic steatosis, supporting the potential therapeutic role of Nrg4 in NAFLD (Wang et al., 2014). These interesting findings demonstrated that Nrg4 deletion can lead to hepatic steatosis by inducing abnormally increased hepatic lipogenesis and that increased Nrg4 expression may be a therapeutic target for NAFLD. Supplementation of n-3 polyunsaturated fatty acids (n-3 PUFAs) to pup rats from postnatal week three to week 13 was revealed to increase Nrg4 and UCP1 mRNA levels in adipose tissue and improve NAFLD (Yang et al., 2021). These effects can be abolished by peroxisome proliferator-activated receptor gamma (PPARG) antagonist, suggesting that n-3 PUFAs may affect adipose tissue browning through PPARG and thus improve NAFLD (Yang et al., 2021).

Autophagy has a key role in the pathogenesis of NAFLD, of which the adenosine monophosphate-activated protein kinase (AMPK)/mTOR pathway represents a key player (Wang et al., 2019b; Filali-Mouncef et al., 2022). A study found that autophagy was downregulated in NAFLD in both *in vivo* and *in vitro* models, while Nrg4 upregulated autophagy through the AMPK/m TOR pathway thus partially contributing to the decrease in lipid accumulation in the liver (Zhu et al., 2020).

The pathogenesis of NASH is closely involved with cell death, tissue inflammation and fibrosis in the liver and hepatic steatosis can progress to NASH following tissue homeostasis disruption (Schuster et al., 2018; Sookoian and Pirola, 2018). Guo et al. (Guo et al., 2017) demonstrated that Nrg4 is a novel endocrine checkpoint in the progression from hepatic steatosis to NASH. Nrg4 mRNA levels were significantly reduced in the adipose tissue of NASH mice, and Nrg4 knockout mice exhibited more severe liver fibrosis and inflammatory infiltration; interestingly, Nrg4 inhibition did not reduce steatosis in NASH mice (Guo et al., 2017). In contrast, Nrg4 transgenic NASH mice manifested attenuated severity of liver inflammation and fibrosis (Guo et al., 2017). Further *in vitro* studies showed that cellular FADD-like IL-1β-converting enzyme-inhibitory protein (c-FLIP), a novel apoptosis inhibitory protein, mediated the protective effect of Nrg4 on hepatocytes (Guo et al., 2017). Nrg4 exerted protective effects against hepatocyte death under stress by reducing ubiquitination and proteasomal degradation of c-FLIP, thereby regulating the post-translational protein levels of c-FLIP (Guo et al., 2017). Liver-specific overexpression of c-FLIP in Nrg4 knockout NASH mice recovered inflammation and fibrosis levels, demonstrating the critical role of c-FLIP in the Nrg4-mediated progression of steatosis to NASH (Guo et al., 2017). In another study, dietary intervention and bariatric surgery restored decreased Nrg4 levels and improved inflammation status in diet-induced NASH mice (De Munck TJIXu et al., 2020). These findings robustly suggested critical protective roles for Nrg4 in NASH progression that are primarily concerned with hepatocyte death and thus ameliorating inflammation and fibrosis, independent of lipid metabolism.

**FIGURE 4:**
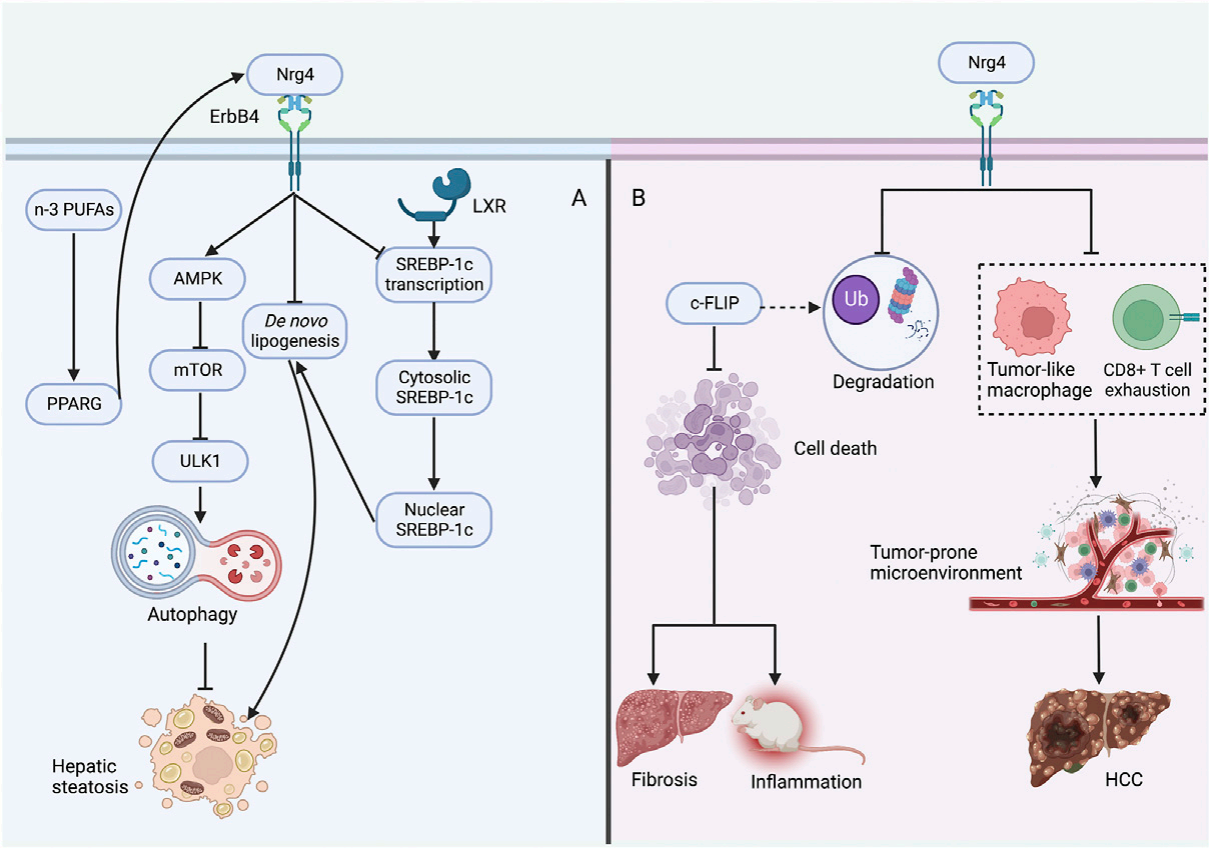
Protective role of Nrg4 in hepatic steatosis and NASH **(A)** Nrg4 specifically binds to the hepatic ErbB4 receptor, increases autophagy through the AMPK/mTOR pathway to improve steatosis, and can also reduce lipogenesis by decreasing the expression of genes involved in *de novo* adipogenesis. Nrg4 can reduce lipogenesis by inhibiting the expression of SREBP-1c. Supplementation of n-3 PUFAs may improve NAFLD by increasing Nrg4 expression through PPARG. **(B)** Nrg4 reduces ubiquitination and proteasomal degradation of c-FLIP to regulate its expression, which can reduce fibrosis and inflammation by reducing hepatocyte death. Alternatively, Nrg4 may prevent HCC development by alleviating the tumor-prone microenvironment in NASH. Abbreviations: Nrg4, neuregulin four; NASH, non-alcoholic steatohepatitis; AMPK, adenosine monophosphate-activated protein kinase; mTOR, mechanistic target of rapamycin; SREBP-1c, sterol regulatory element binding protein-1c; ULK1, unc-51-like kinase one; LXR, liver-X receptor; PPARG, peroxisome proliferator-activated receptor gamma; n-3 PUFAs, n-3 polyunsaturated fatty acids; c-FLIP, cellular FADD-like IL-1β-converting enzyme-inhibitory protein; HCC, hepatocellular carcinoma.

A recent study by Zhang et al. (Zhang et al., 2022b) demonstrated that the Nrg4 endocrine axis can arrest the development of HCC by inhibiting the tumor-promoting microenvironment in NASH. It was demonstrated that there was a strong induction of tumor-like macrophages and exhaustion of CD8^+^ T cells (cytotoxic T lymphocytes) in NASH, and Nrg4 knockout mice were shown to exacerbate the deregulation of the hepatic immune microenvironment (Zhang et al., 2022b). Further construction of human Nrg4-Fc fusion protein revealed remarkably reduced induction of NASH-associated macrophages and T-cell depletion-related gene expression in the liver, demonstrating its potent inhibitory effect on the tumor-prone microenvironment in mice (Zhang et al., 2022b). Together, these solid findings demonstrate that Nrg4 serves as an immune checkpoint involved in the crosstalk of the pro-oncogenic microenvironment in NASH and can protect against HCC through profound immune reprogramming (Figure 4).

Several clinical studies have further demonstrated the association between Nrg4 expression and NAFLD. A single-center case-control study enrolling 174 subjects found that serum Nrg4 levels were significantly decreased in patients with NAFLD compared to healthy controls and were markedly associated with NAFLD, although not with the severity of NAFLD as assessed by ultrasound (Dai et al., 2015). Another study including 123 children with obesity found that circulating Nrg4 levels were negatively and significantly associated with NAFLD and other metabolic-related parameters (Wang et al., 2019c). A study included patients with NAFLD diagnosed by ultrasound also found that circulating Nrg4 levels were negatively associated with NAFLD, where BMI, waist circumference, high-density lipoprotein cholesterol (HDL-C), and homeostatic model assessment for insulin resistance (HOMA-IR) were independently associated with Nrg4 (Tutunchi et al., 2021). For each standard deviation increase in Nrg4 serum levels, a 41% reduction in the odds of NAFLD was identified after adjusting for confounding factors (Tutunchi et al., 2021). However, another study illustrated that serum Nrg4 levels were not associated with NAFLD, hepatic fat fraction, and NAFLD-associated fibrosis in patients where chemical shift magnetic resonance imaging and transient elastography were used to diagnose NAFLD and associated fibrosis, respectively (De Munck TJIBoesch et al., 2021). The different findings of these studies may be due to the heterogeneity of the study populations and methods, thus this is further evidence that the role of Nrg4 in the clinical setting may be influenced by multiple factors (De Munck TJIBoesch et al., 2021). The limitations of these studies are the lack of representative data and the nature of observational studies to determine a causal relationship between Nrg4 and NAFLD, and larger well-designed studies are needed to validate this relationship in the future. A recent study identified two rare missense mutations in *NRG4*, Nrg4 E47Q, and Nrg4 R44H, by whole-exome sequencing of 224 subjects with obesity and exome genotyping of 2,388 participants from the Shanghai Obesity Study (Li et al., 2021). Nrg4 E47Q was demonstrated to potentiate the protective effects of Nrg4 against NAFLD in animal models by negatively regulating *de novo* lipogenesis, whereas Nrg4 R44H was deprived of these effects (Li et al., 2021). This indicated that genetic variation in *NRG4* in the population can lead to enhanced or impaired metabolic benefits, which may result in different clinical outcomes.

### Nrg4 and diabetes

Gluconeogenic activity is significantly impaired in diabetes (Sharma and Tiwari, 2021), and hepatic Nrg4 was revealed in a study to play a crucial role in the regulation of gluconeogenesis in mice (Zhang et al., 2019), suggesting that Nrg4 may serve as a potential target in diabetes. The relationship between circulating Nrg4 levels and diabetes has been reported in extensive observational studies with inconsistent findings (Wang et al., 2019d). Several studies have identified elevated serum Nrg4 levels in patients with diabetes (Kang et al., 2016; Chen et al., 2017b; Kurek Eken et al., 2018; Kocak et al., 2019), while other studies have associated decreased serum Nrg4 levels with an increased risk of diabetes (Zhang et al., 2017; Kralisch et al., 2018; Zhang et al., 2021; Attique et al., 2022; Li et al., 2022). (Table 1) Several studies have shown that serum Nrg4 values were significantly elevated in patients with type 2 diabetes mellitus (T2DM) compared to healthy controls and were positively correlated with serum glucose levels (Kang et al., 2016; Chen et al., 2017b; Kocak et al., 2019), while Zhang et al. (Zhang et al., 2017) suggested that Nrg4 levels were significantly decreased in newly diagnosed T2DM. These conflicting findings may be explained by several aspects. Firstly, ethnic differences in the subjects included in each study may be a source of discrepancy (Mikhail et al., 2021), as the patients were from the same country or region, which may lead to heterogeneity in their clinical characteristics. Second, the elevated serum Nrg4 levels in T2DM patients may represent a compensatory response to reduced Nrg4 expression in adipose tissue or impaired Nrg4/ErbB signaling pathway mediated by receptor resistance (Kang et al., 2016; Chen et al., 2017b). Furthermore, these studies have generally been observational studies with small samples and therefore lack representativeness and were unable to determine the causal relationship between serum Nrg4 levels and T2DM. Other studies have focused on the association of gestational diabetes mellitus (GDM) with serum Nrg4 levels, and inconsistent findings also have been reported. Eken et al. (Kurek Eken et al., 2018) concluded that circulating Nrg4 concentrations were increased in GDM patients, while other studies (Kralisch et al., 2018; Zhang et al., 2021; Attique et al., 2022; Li et al., 2022) have observed the opposite trend. Similarly, this difference may be attributed to differences in clinical characteristics, ethnicity, and enzyme-linked immunosorbent assay techniques (methods of detecting serum Nrg4).

**TABLE 1:** Association of serum Nrg4 levels with diabetes mellitus.

References	Country, year	Study cohort	Study design	Nrg4 measurement	Conclusion
Kang et al., (2016)	South Korea, 2016	57 patients with newly diagnosed T2DM and 59 controls without diabetes	Single center case-control study	ELISA	Serum Nrg4 level was positively correlated with fasting plasma glucose level, HOMA-IR, and fasting triglyceride level
Chen et al., (2017b)	China, 2017	83 subjects with normal glucose tolerance, 129 with prediabetes and 96 with diabetes who were age-, sex- and BMI-matched	Case-control study	ELISA	Serum Nrg4 level was elevated in patients with prediabetes and diabetes compared to controls
Kocak et al., (2019)	Turkey, 2019	100 patients with T2DM and 50 controls	Single center cross-sectional cohort study	ELISA	There were significant differences in Nrg4 levels among the poorly controlled T2DM, the well-controlled T2DM, and the controls; Nrg4 was significantly correlated with fasting plasma glucose; a .1-point increase in Nrg4 levels was associated with a 4.4-fold increase of T2DM presence
Kurek Eken et al., (2018)	Turkey, 2018	63 women with GDM and 64 healthy pregnant women matched for age, BMI, and gestational age	Single center prospective cross-sectional study	ELISA	Serum Nrg4 level was significantly higher in the GDM group compared to the controls
Zhang et al., (2017)	China, 2017	103 newly diagnosed T2DM patients and 129 age-, sex-, and BMI-matched controls	Single center cross-sectional study	ELISA	Serum Nrg4 level decreased in newly diagnosed T2DM patients compared to controls
Kralisch et al., (2018)	Germany, 2018	74 women with GDM and 74 pregnant women with normal glucose tolerance	Single center cross-sectional study	ELISA	Serum Nrg4 level was significantly lower in women with GDM; Nrg4 was positively correlated with irisin during pregnancy
Zhang et al., (2021)	China, 2021	36 patients with GDM and 38 age-, gestational age-, and BMI-matched controls	Single center cross-sectional study	ELISA	Serum Nrg4 was significantly lower in patients with GDM; serum Nrg4 was negatively correlated with fasting glucose, HOMA-IR, IL-6, leptin, TNF-α and MCP-1, and positively correlated with HDL-C
Attique et al., (2022)	Pakistan, 2022	37 patients with GDM and 47 healthy pregnant women	Single center cross-sectional study	ELISA	Serum Nrg4 level was significantly lower in GDM subjects; insulin and HOMA-IR were significantly correlated with Nrg4
Li et al., (2022)	China, 2022	58 patients with GDM and 60 pregnant women without GDM	Single center cross-sectional study	ELISA	Serum Nrg4 level was reduced in GDM patients

Nrg4, neuregulin four; T2DM, type 2 diabetes mellitus; ELISA, enzyme-linked immunosorbent assay; HOMA-IR, homeostatic model assessment for insulin resistance; BMI, body mass index; GDM, gestational diabetes mellitus; IL-6, interleukin-6; TNFα, tumor necrosis factor alpha; MCP-1, monocyte chemoattractant protein-1; HDL-C, high-density lipoprotein cholesterol.

The association of serum Nrg4 levels with diabetic complications has also been noted. Serum Nrg4 levels were negatively correlated with inflammatory marker high-sensitivity C-reactive protein (hs-CRP) levels (Yan et al., 2017) and the development of MetS (Yan et al., 2018) in patients with diabetes, suggesting a potential relevance of reduced circulating levels of Nrg4 in T2DM in the pathogenesis of inflammation and metabolic dysregulation. A cross-sectional study including 132 Chinese patients with T2DM demonstrated that serum Nrg4 levels were reduced in diabetic peripheral neuropathy (DPN) and significantly associated with its development, further implicating the anti-inflammatory and metabolic benefits of Nrg4 in T2DM (Yan et al., 2019). Another study (Yan et al., 2020) suggested that serum Nrg4 in DPN patients was independently and positively correlated with 25-hydroxy vitamin D, a hormone that modulates neurotrophic factors and exerts neuronal trophic effects, which seemed to indicate that Nrg4 exerted beneficial effects by interacting with this vitamin. Another study found that serum Nrg4 levels could be a promising predictive biomarker of diabetic microvascular complications with a sensitivity of 82.1% (Kocak et al., 2020).

A preclinical study found that Nrg4 expression was downregulated in rats with diabetic nephropathy and exerted therapeutic effects *via* TNF receptor one signaling (Shi et al., 2019). Another study similarly demonstrated decreased Nrg4 mRNA expression in adipose tissue in mice with diabetic kidney disease and significantly decreased circulating Nrg4 levels in human end-stage kidney disease, suggesting a potential protective effect of Nrg4 on kidney function (Kralisch et al., 2019). A recent study (Wang et al., 2022b) demonstrated that Nrg4 treatment could upregulate decreased autophagy activity *via* the AMPK/mTOR pathway in type 1 diabetic mice, and further revealed that it attenuated myocardial damage caused by diabetic cardiomyopathy *in vivo* and *in vitro*, suggesting that Nrg4 may exert a protective effect as a potent regulator of autophagy. These preclinical and clinical studies highlight Nrg4 as a promising therapeutic target and clinical predictive marker for diabetes and its complications.

### Nrg4 and other metabolic disorders

Polycystic ovary syndrome (PCOS) is a polygenic, multifactorial endocrine disorder commonly seen in reproductive-aged women, usually associated with IR and glycolipid metabolic disturbances, and at significantly increased risk for T2DM, MetS, and NAFLD (Azziz, 2018). A case-control study including 40 women with PCOS and 40 age- and BMI-matched controls found that serum Nrg4 levels were significantly higher in patients with PCOS than in controls and positively correlated with HOMA-IR, hs-CRP, and circulating insulin (Temur et al., 2017). Another study observed significantly higher Nrg4 levels in obese women with PCOS than in normal-weight women with PCOS, and multivariate analysis suggested that BMI was an independent factor impacting Nrg4 expression in PCOS patients (Kurek Eken et al., 2019). These findings may be interpreted by increased compensatory expression associated with metabolic dysregulation such as IR, leptin resistance, or the presence of Nrg4 resistance status in PCOS patients. Serum Nrg4 levels in adolescent girls with obesity and PCOS were significantly downregulated by a 1-year weight intervention, which remarkably improved clinical symptoms (Cao and Hu, 2021).

Cardiovascular diseases are strongly associated with metabolic derangement conditions (Karlstaedt et al., 2022). Nrg4 has been identified as a potential cardioprotective protein, that is, altered in multiple CVDs (Geissler et al., 2020). It was revealed that the liver can upregulate cardioprotective proteins such as Nrg4, thus protecting the heart under experimental myocardial ischemia (Liu et al., 2012). In the clinical setting, circulating Nrg4 has similarly exhibited cardioprotective effects. Circulating Nrg4 levels were negatively associated with subclinical atherosclerosis in adults with obesity (Jiang et al., 2016). Circulating Nrg4 levels were inversely correlated with the presence and severity of coronary artery disease (CAD) and could be employed as a highly specific marker for identifying CAD presence (Tian et al., 2019). This protective effect may be exerted through interaction with IL-9, a cytokine that may be involved in its pathogenesis, as Nrg4 was negatively correlated with IL-9 (Farshidi et al., 2021). Serum Nrg4 levels were significantly lower in acute coronary syndrome (ACS) and negatively correlated with HDL-C contents, a well-known beneficial lipoprotein, suggesting a possible association of serum Nrg4 levels with ACS prevalence (Rahimzadeh et al., 2020).

Nrg4 treatment was revealed to delay the progression of osteoarthritis by exerting anti-inflammatory and anti-apoptotic effects on chondrocytes *in vivo* and *in vitro* and alleviating extracellular matrix degradation *in vitro* (Shi et al., 2022). A study identified significantly increased serum Nrg4 levels in patients with hyperthyroidism and reduced levels after recovery of thyroid function (Li et al., 2019). Further experiments showed that thyroid hormones increased Nrg4 expression in the liver and WAT (Li et al., 2019).

## Conclusion

Nrg4 as an adipokine enriched in BAT has been revealed to potentiate BAT-related thermogenesis by modulating sympathetic innervation of adipose tissue and has demonstrated potential as a therapeutic target in multiple metabolic disorders. In preclinical studies, Nrg4 as a novel endocrine checkpoint exerted protective effects against various metabolic diseases such as IR, obesity, NAFLD, and diabetes; while discrepancies were noted in clinical investigations, more well-designed studies with large samples are needed in the future to address the heterogeneity and inconsistency. Nrg4 is partially involved in lifestyle interventions such as training, bariatric surgery and certain medications for improved metabolic dysregulation. Therefore, Nrg4 may represent a future endocrine target for the treatment of metabolic disorders.
